# Systematic composition, species diversity and plant chorology of Wadi Shuwaib: A typical desert wadi in the eastern region of United Arab Emirates (UAE)

**DOI:** 10.3897/BDJ.13.e159155

**Published:** 2025-07-02

**Authors:** Sabitha Sakkir, Maher Kabshawi, Nuri Asmita, Salama Almansoori, Hassan Alahbabi

**Affiliations:** 1 Environment Agency - Abu Dhabi, Abu Dhabi, United Arab Emirates Environment Agency - Abu Dhabi Abu Dhabi United Arab Emirates

**Keywords:** angiosperms, wadis and mountains, flora, lifeform, new records, threatened species

## Abstract

The study analyses the floristic diversity, life span, life form and phytogeographic affinities of the flora of Wadi Shuwaib, a desert wadi in the eastern region of the United Arab Emirates. A total of 123 species of vascular plants were recorded belonging to 95 genera and 35 families. The most represented families were Fabaceae, Asteraceae, Poaceae and Brassicaceae. Eleven families were represented by a single species each and 77 (81.9 %) genera were monotypic. Eight species have been recorded for the first time in Abu Dhabi Emirate and represent 1.8% increase in the flora of Abu Dhabi. The recorded flora consists of 50.40% perennials and 49.59% annuals. Therophytes and Chamaephytes were the most frequent lifeforms. Phytogeographical analysis revealed that the biregional elements of the Saharo-Arabian/Sudano-Zambezian chorotype are the most dominant chorotypes (22.76%), forming approximately one fourth of the floristic structure in Wadi Shuwaib. The new additions to the local flora of the region indicate that the Wadi Shuwaib Region and the country need further thorough botanical exploration and documentation which would help in adding new species to the flora of UAE.

## Introduction

The United Arab Emirates (UAE), which occupies an area of about 83,600 km^2^ is characterised by different ecosystems and diversity of plant species. The recently published report recognises a total of 598 native plant species for UAE (Allen 2021). UAE’s mountain regions occupy less than 5% of its surface area and are home to more than 50% of its native plants (Feulner 2023). In the UAE, the mountain ecosystem, mainly the Hajar Mountain occupies Fujairah Emirate, Ras Al Khaimah, parts of Sharjah, Dubai and Ajman enclaves of Hatta and Masfut, respectively (Feulner 2011, Feulner 2023). Jabal Hafit, the foreland ridge of the Hajar Mountains is in the Al Ain Region of the Abu Dhabi Emirate and support the highest species diversity (Sakkir and Brown 2014) in the Abu Dhabi Emirate.

The UAE is characterised by a hot and dry climate throughout most of the year and is classified as hyper-arid area according to the UNEP classification of drylands (Middleton and Thomas 1997). Despite its aridity, the UAE has a relatively rich flora, especially in regions that retain some moisture during the precipitation months, including some microhabitats of mountainous regions, wadis and silt pans in front of dams (Mahmoud 2018).

Mountains and wadis are a heterogeneous ecosystem characterised by cooler temperatures and more humid microhabitats than other ecosystems from Abu Dhabi Emirate (Javed 2023). Wadis (the Arabic term referring to a valley) are unique intrazonal landscapes in arid and semi-arid regions of the world and represent one of the most prominent desert landforms, which exhibits physiographic irregularities that lead to parallel variations in distribution of plant species (Kassas and Girgis 1964). As these wadis are drainage systems to collect water from the nearby mountains, the vegetation in these wadis is richer when compared to other desert ecosystems. Vegetation of the wadis can usually be destroyed by two factors, such as torrents and grazing. The influence of torrents on plants is partly mechanical, destroying and uprooting the plants and partly erosive removing the soil (Kassas and Girgis 1964). The wadi vegetation is also subjected to grazing and grasses like *Panicumantidotale* Retz. and woody trees like *Prosopiscineraria* (L.) Druce are liable to be cut as a source as fodder.

The wadis of the UAE exhibit significant floristic diversity, characterised by a unique assemblage of plant species adapted to the arid environment. Studies conducted over the years, including those by Brown and Sakkir 2004, El-Keblawy et al. 2016 and Mahmoud 2018 have highlighted the ecological significance of these ecosystems. The rich vegetation found in wadis supports a range of endemic and native species, contributing to biodiversity conservation in the region. The establishment, growth, regeneration and distribution of the plant communities in the wadis are controlled by many factors, such as geographical position, physiographic features and human impacts (Shaltout and El-Sheikh 2002, Kürschner and Neef 2011, Alatar et al. 2012).

The aim of this manuscript is to analyse the vegetation of Wadi Shuwaib in terms of the floristic composition, lifeform and chorotype which, in turn, help to assess the floristic wealth of the region and understanding the basic aspects of biology such as speciation, isolation, endemism and evolution. There are no earlier reports on the flora of Wadi Shuwaib, which leads us to recognise that more floristic work is needed to fill the gaps in our understanding of the flora. The present study aims to identify and document the floristic diversity, life span, lifeforms and phytogeographic relationships and present a checklist of the flora of the wadi and thereby updating the checklist of the flora of Abu Dhabi Emirate and the UAE.

## Materials and Methods

### Study area

**Figure 1.**
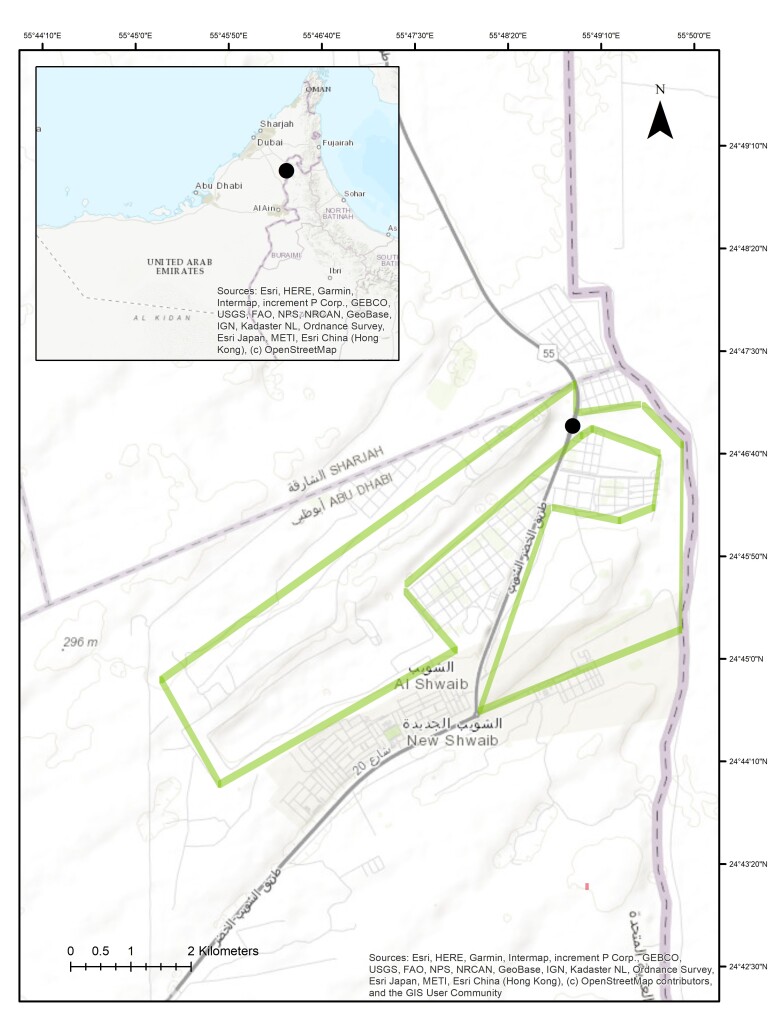
Location map of the study area (inset map of the UAE).

Wadi Shuwaib is located on the eastern part of the United Arab Emirates, in the Abu Dhabi Emirate, specifically in the Al Ain Region, which borders the Sultanate of Oman. It is situated on the Hajar Mountains, the largest mountain range that stretches across the borders between UAE and Oman. A dam was built to store the runoff rainwater from the surrounding mountain. The surrounding mountains receive significant rainfall during the winter months which collects in the wadis. The scope of the study was confined to the section of the Wadi Shuwaib within the UAE while a significant portion of the wadi lies within the region of Oman. (Fig. 1).

**Figure 2.**
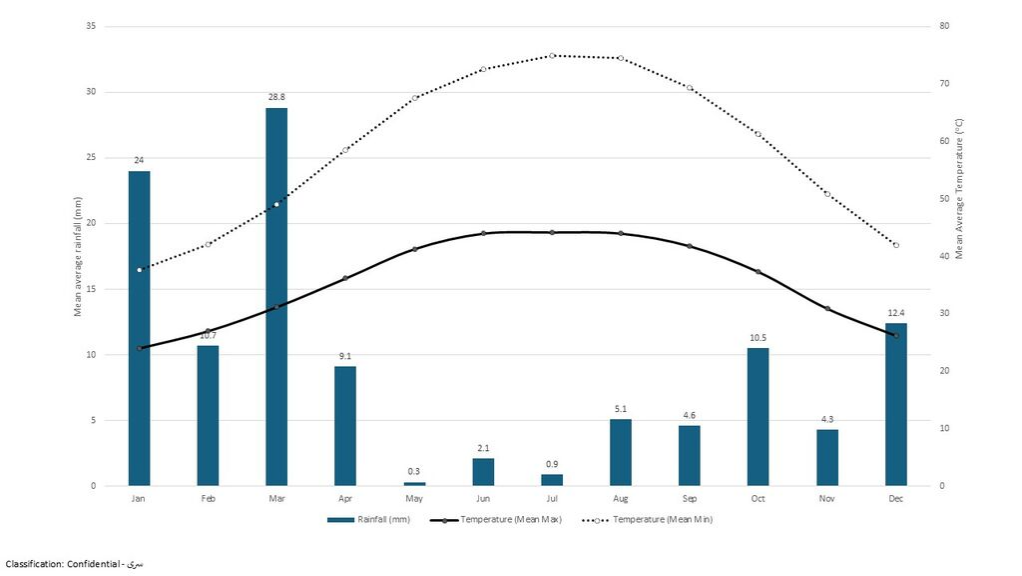
Temperature (^o^C) at Shuwaib Weather station during 2022-2024. Sources: https://www.ncm.gov.ae/services/climate-reports-yearly?lang=en.

The UAE’s Mountain regions receive mean annual rainfall of 160–190 mm and are, therefore, routinely classified as arid overall, although not hyper-arid (Feulner 2024). The study area is situated on the eastern part of the UAE. January is the coldest month with the lowest average temperature (13.7^o^C) and the hottest month is July with the highest average temperature of 44.2^o^C. The maximum rainfall (28.8 mm) was estimated during the month of March and the minimum of 0.3 mm during May (Fig. 2). (National Centre for Meteorology, Al Shuwaib weather station, Elevation: 292 m, Latitude: 24^o^ 46’41’’ N, Longitude: 55^o^47’58’’ E).

### Plant collection and Taxonomic identification

The species checklist presented in this study is the result of a floristic survey conducted during a period of 2023–2024. The field attributes were collected using an iPad-based IOS Collector App, which directly syncs with the plant database of the Environment Agency – Abu Dhabi (EAD). The dataset of all the records collected for the present work was deposited in a common data repository (Sakkir 2025). Plant specimens were collected and identified and named according to Jongbloed (2003). Plant lifeforms along with the life span were determined (Raunkiaer 1934) and phytogeographical affinities of the surveyed species were defined (Agnew and Zohary 1974). Specimens were dried and deposited in the Herbarium of EAD.

## Results

### Floristic composition

A total of 123 taxa of vascular plants were recorded from the study area, belonging to 95 genera and 35 families. Amongst them, eight species have been recorded for the first time in Abu Dhabi Emirate and represent new additions to the flora of Abu Dhabi (Appendix 1). Four species fall under the threatened category, of which *Volutariasinaica* (DC.) Wagenitz listed as threatened in the UAE National Red List of species, while *Vachelliatortilis* (Forssk.) Galasso & Banfi,*Prosopiscineraria* (L.) Druce and *Lyciumshawii* Roem. and Schult were listed as threatened in Abu Dhabi Red List of species (Javed et al. 2020). The alien taxa *Verbesinaencelioides* (Cav.) A.Gray, which is an annual and grows primarily in the temperate biome (Royal Botanic Gardens, Kew 2025) was also recorded for the first time in Abu Dhabi Emirate.

All the species recorded were Angiosperms, of which 33 families (94.28%) were dicotyledons with 109 taxa (88.61%), while monocotyledons were represented by two families, Asphodelaceae with a single taxon, *Asphodelustenuifolius* Cav. and Poaceae with 13 (10.56%) taxa.

**Figure 3.**
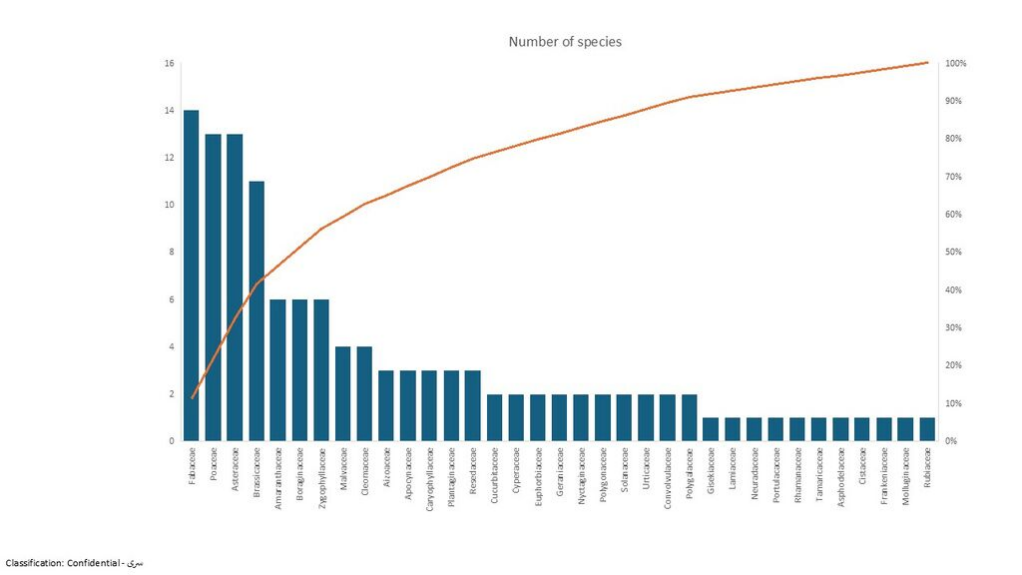
Histogram of the floristic composition of the 29 families recorded in Wadi Shuwaib.

The most species rich families were Fabaceae (14 species = 11.38%), Asteraceae (13 species = 10.56%), Poaceae (13 species = 10.56%) and Brassicaceae (11 species = 8.94%). Amaranthaceae, Boraginaceae and Zygophyllaceae were represented by six species (4.87%) each, while Malvaceae and Cleomaceae were represented by four species each (3.25%). The families Aizoaceae, Apocynaceae, Caryophyllaceae, Plantaginaceae and Resedaceae were represented by three species each (2.43%). Ten families were represented by two species and 11 families were represented by a single species. The largest families in terms of the number of genera were Fabaceae (12 genera), Brassicaceae (11 genera), Poaceae (10) genera and Asteraceae (10 genera) (Fig. 3).

The genera with largest number of species were *Cleome* L., *Heliotropium* Tourn. ex L., *Tribulus* L. and *Launaea* Cass. with four species each, followed by *Cenchrus* L. and *Plantago* L. with three species each. Twelve genera were represented by two species each, while 77 genera were represented by a single species. According to the lifeform, the perennials were represented by 62 species (50.40%) and annuals were represented by 61 species (49.59%) (Table 2).

### Lifeform spectra

**Figure 4.**
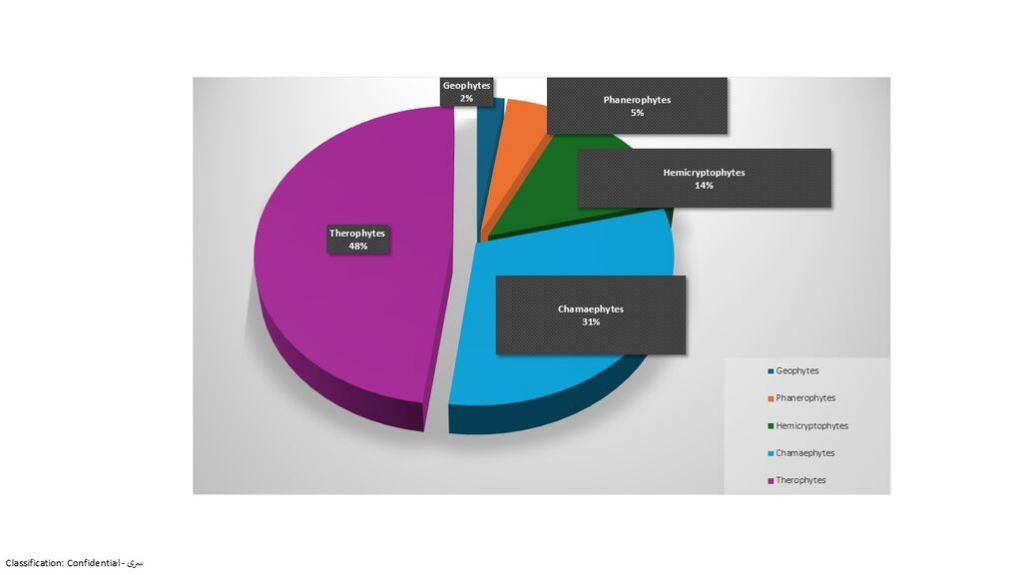
Lifeform spectra of the plant species recorded from Wadi Shuwaib.

The studied flora falls under five major lifeforms. Therophytes were the most frequent lifeforms (59 species = 47.97%) followed by Chamaephytes (38 species = 30.89%), Hemicryptophytes (17 species = 13.82%), Phanerophytes (6 species = 4.88%) and Geophytes (3 species = 2.44%) (Table 2, Fig. 4).

### Chorological affinities

 Chorological analysis of the 123 plant species recorded in this study has been classified into three major phytogeographical groups: monoregional, biregional and pluriregional.

A total of 26 species representing 21.14% of the total number of recorded species were monoregional taxa of different affinities. The monoregional elements fall under four main chorotypes: Saharo-Arabian taxa (17 species forming 13.82% of recorded species), Sudano-Zambezian taxa (4 species forming 3.25% of recorded species) and Saharo- Sindian taxa (3 species forming 2.44% of recorded species). Two Irano-Turanian taxa were recorded in the study area (*Centaureapseudosinaica* Czerep and *Paronychiaarabica* (L.) DC.) representing 1.63% of the surveyed flora.

The biregional elements has the highest representation (49 species = 39.84%) with different affinities. The biregionals falls under eight main chorotypes: the Saharo-Arabian/Sudano-Zambezian chorotypes together have the highest number of species (28 species) representing 22.76% of the total flora, followed by the Irano-Turanian/Saharo-Arabian, Irano-Turanian/Saharo-Sindian, Saharo-Sindian/Sudano-Zambezian and Mediterranean/Saharo-Arabian Region represented by four species each (3.35%). The Irano-Turanian / Mediterranean Region and were represented by three species (2.44%). The lowest number of species was recorded for the Mediterranean/Saharo-Sindian (*Erucariahispanica* Druce) Mediterranean/ Sudano-Zambezian (*Tribulusterrestris* L.) represented by a single species each.

The pluriregional elements were represented by a total of 34 species (17.07%) of different affinities. The pluriregional elements falls under nine different chorotypes: Irano-Turanian/Saharo-Arabian/Sudano-Zambezian (13 species forming 10.57% of recorded species) has the highest number of species in the pluriregional chorotype. The Irano-Turanian/Mediterranean/Saharo-Sindian/Sudano-Zambezian, Irano-Turanian/Mediterranean/Saharo-Sindian/Sudano-Zambezian and Irano-Turanian/Mediterranean/Saharo-Sindian were represented by four species each (3.25%). Irano-Turanian/Mediterranean/Saharo-Arabian, Irano-Turanian/Saharo-Sindian/Sudano-Zambezian, Mediterranean/Saharo-Arabian/Sudano-Zambezian and Irano-Turanian/Mediterranean/Tropical Region were represented by two species each (1.63%), while Mediterranean/Saharo-Sindian/Sudano-Zambezian were represented by a single species (*Sisymbriumirio* L.). The remaining 12 species were distributed amongst Cosmopolitan (6 species = 4.88%), Pantropical (3 species = 2.44%), Palaeotropical (2 species = 1.63%) and Neotropical (1 species = 0.81%) chorotypes. (Table 1).

## Discussion

The mountain and the associated wadi ecosystems in the UAE are of great floristic and ecological importance. The present study reveals that the floristic composition of Wadi Shuwaib is highly diverse. The diversity may be due to various environmental factors which are favourable for most of the species recorded. In the current study, the floristic analysis of vascular plant species from the study area includes 123 species belonging to 95 genera and 35 families (Table 2). This is thought to be mainly due to the presence of wadi habitat where runoff water collects and provide sufficient moisture and suitable conditions for the plant growth. As water is the most limiting factor for plant growth and survival in arid regions, the wadi bed provides the most favourable conditions for plant growth, especially for plants that have greater water requirements (El-Keblawy et al. 2016). These areas are also liable to wash-outs during flash floods.

The floristic diversity of wadi ecosystems in the UAE has been extensively documented through various studies by Brown and Sakkir 2004,Mousa and Fawzi 2009,Mousa 2011, El-Keblawy et al. 2016, and Mahmoud 2018 which highlighted the floristic diversity of wadi ecosystems in the UAE. All these studies have shown that wadi vegetation is diverse and supports a range of endemic and native species, contributing to biodiversity conservation in the region. The present study conducted at Wadi Shuwaib further supports these findings, highlighting the significant floristic diversity present within this specific wadi.

The floristic survey showed that most species rich families were Fabaceae (14 species), Asteraceae (13 species), Poaceae (13 species) and Brassicaceae (11 species). These results confirmed to those of Mahmoud 2018and Brown and Feulner 2024. This can be attributed to their efficient germination strategies, seed dispersal capabilities, migration efficiency and wide ecological range of tolerance, in addition to local conditions of water availability (Brown and Feulner 2024). The present survey revealed also that only few families are floristically rich and the floristic composition of Wadi Shuwaib exhibited a high degree of monotypism, where 77 genera were represented by a single species. This may be because flora of an area, in which species are distributed amongst various genera, families or other higher ranks, exhibit greater genomic information and phylogenetic diversity than that where most species belong to the same genus or concentrated into fewer higher ranks (Khedr 2002).

The vegetation consisted of almost equal representation of the annual and perennial species. This could be due to the rainfall events which provides a better chance for the appearance of a considerable number of annuals, which have high reproductive capability and have the capacity to dominate the ecosystems, thereby giving a characteristic physiognomy to their vegetation (Shaltout 2010). Perennial plants are adapted to the extreme climatic conditions and they have different adaptations and survival strategies to cope with the harsh climatic conditions which are discussed in detail by Brown and Feulner (2024). The presence of synanthropic species such as *Bassiaeriophora*, *Cynodondactylon* and *Tamarix* sp. indicated the presence of human impact in the area.

The UAE has no nationally endemic plant species, but seven species found in the UAE are considered endemic to the mountains of the UAE and northern Oman (Feulner 2016). *Echinopserinaceus* which is endemic to the mountains of the UAE and northern Oman was recorded from the study area. Moreover, four species recorded fall under the threatened category both locally and nationally. The presence of endemics and threatened species in the study area indicates special ecological and biogeographic importance of the area.

The distribution of lifeforms is closely related to topography and landform (Kassas and Girgis 1964, Agnew and Zohary 1974, Evenari et al. 1986, Shaltout 2010). In the present study, the composition of lifeforms expresses a typical desert flora, most species being therophytes and chamaephytes. These results agree with the spectra of vegetation in other parts of UAE (Mahmoud 2018). Therophytes are characteristic of desert climate (Malik 2007). Therefore, the dominance of therophytes in the study area may be an outcome of the harsh weather and high aridity in the region. The high percentage of chamaephytes may be related to their ability to resist to the drought, salinity and sand accumulation (Bana 2002).

According to Feulner (2011), the mountain areas of the UAE are a meeting point for floral elements from the Saharo-Arabian, Nubo-Sindhian, Irano-Turanian and Mediterranean Regions. Chorological analysis of the floristic data from the study area revealed that the biregional Saharo-Arabian/Sudano-Zambezian chorotypes together have the highest number of species, which are good indicators of a desert environment. Typical representatives of the Saharo-Arabian Region such as *Anastaticahierochuntica*, *Haloxylonsalicornicum*, *Neuradaprocumbens*, *Savignyaparviflora* and *Stipagrostis* spp. were recorded from the study area. Some of the characteristic species of Sudanian Region present in the study area includes *Acaciatortilis*, *Calotropisprocera*, *Leptadeniapyrotechnica*, *Panicumturgidum*, *Pennisetumdivisum* etc. The combination of different phytochoria with uneven numbers of species can be attributed to factors such as diversity of habitats, topography, water availability and the capability of certain taxa to penetrate the study area from different adjacent phytogeographical regions (Abbas et al. 2020).

The Emirate of Abu Dhabi is home to around 436 species of native plants distributed in diverse habitats (Dhaheri 2017). The present survey also found seven species that had not been recorded before in the flora of the Abu Dhabi. The presence of new records of in the wadi can be attributed to the following factors: (i) The unique geographical location of Wadi Shuwaib; (ii) its proximity to the Hajar Mountain range, which trigger most of the rainfall descending on this land; (iii) the presence of the dam; and (iv) The rich and diverse flora of the study area owing to the combination of Saharo-Arabian/Sudano-Zambezian species. There was a widespread distribution of the alien taxa, *Verbesinaencelioides* in the wadis. As Wadi Shuwaib lies to the border with Oman, the major invasions of the flora of the area are due to intentional or unintentional actions of humans, animals, birds and, to some extent, due to traffic across the borders.

The combination of extreme summer temperatures, low annual rainfall and the unpredictability of rainfall poses challenges for the native flora and fauna of the UAE (Feulner 2023). Preserving the unique wadi ecosystems in the UAE is crucial due to their ecological, hydrological and cultural significance. Effective conservation strategies such as habitat protection, systematic species monitoring and infrastructure to manage flash floods can help maintain biodiversity, protect water resources and support sustainable development in such ecosystems. A total of eight species being new additions to the local flora of the region, represents a 1.8% increase on the previously published species list of Abu Dhabi Emirate.

## Conclusion

The new records highlight the ongoing contributions to the botanical understanding of the Emirate and indicate the need for further botanical exploration and documentation which would help in adding species records to the local flora and to the flora of UAE. This also underscores the importance of protecting the natural ecosystems of the country.

## Figures and Tables

**Table 1.** Number of plant species belonging to the main floristic chorotype and their relevant percentage recorded in Wadi Shuwaib. Chorotypes abbreviations: ME: Mediterranean, NEO: Neotropical, PAL: Palaeotropical, PAN: Pantropical, IR-TR: Irano-Turanian, SA-AR: Saharo-Arabian, SA-SI: Saharo-Sindian, SU-ZA: Sudano-Zambezian.

**Chorotype**	**Number of species**	**Percentage (%) of flora**
Cosmopolitan	6	4.88
Neotropical	1	0.81
Palaeotropical	2	1.63
Pantropical	3	2.44
Total	12	9.76
**Monoregional**		
SA-AR	17	13.82
SA-SI	3	2.44
SU-ZA	4	3.25
IR-TR	2	1.63
Total	26	21.14
**Biregional**		
SA-AR+SU-ZA	28	22.76
IR-TR+SA-SI	4	3.25
IR-TR+SA-AR	4	3.25
SA-SI + SU-ZA	4	3.25
ME+SA-AR	4	3.25
IR-TR+ME	3	2.44
ME + SA-SI	1	0.81
ME+SU-ZA	1	0.81
Total	49	39.84
**Pluriregional**		
IR-TR + SA-AR + SU-ZA	13	10.57
IR-TR + ME + SA-AR + SU-ZA	4	3.25
IR-TR + ME + SA-SI +SU-ZA	4	3.25
IR-TR + ME + SA-SI	4	3.25
IR-TR + ME + SA-AR	2	1.63
IR-TR + SA-SI + SU-ZA	2	1.63
ME+SA-AR+SU-ZA	2	1.63
IR-TR+ME+TR	2	1.63
ME + SA-SI + SU-ZA	1	0.81
Total	34	17.07

**Table 2.** Checklist of plant species recorded in Wadi Shuwaib along with their families, life span, lifeform and chorotypes.* New records for Abu Dhabi Emirate.

**Family**	**Taxa**	**Life form**	**Life span**	**Chorotype**
Aizoaceae	*Aizooncanariense* L.	He	Ann	SU-ZA
	*Sesuviumverrucosum* Raf.	He	Per	SA-AR+SU-ZA
	*Zaleyapentandra* (L.) C.Jeffrey	Th	Per	IR-TR + SA-AR + SU-ZA
Amaranthaceae	*Aervajavanica* Juss.	Ch	Ann	SA-AR+SU-ZA
	*Amaranthusgraecizans* L.	Th	Ann	PAL
	*Amaranthusviridis* L.	Th	Ann	COSM
	*Bassiaeriophora* (Schrad.) Asch.	Ch	Per	IR-TR + SA-AR + SU-ZA
	*Caroxylonimbricatum* (Forssk.) Moq.	Ch	Per	SA-AR+SU-ZA
	*Haloxylonsalicornicum* (Moq.) Bunge ex Boiss.	Ch	Per	IR-TR + SA-AR + SU-ZA
Apocynaceae	*Calotropisprocera* (Aiton) Dryand.	Ph	Per	SA-AR+SU-ZA
	*Leptadeniapyrotechnica* (Forssk.) Decne.	Ch	Per	SA-AR+SU-ZA
	*Pergulariatomentosa* L.	Ch	Per	SA-AR+SU-ZA
Asphodelaceae	*Asphodelustenuifolius* Cav.	Th	Ann	ME+SA-AR
Asteraceae	*Atractyliscarduus* (Forssk.) C.Chr.	Th	Per	ME
	*Centaureapseudosinaica* Czerep.	Th	Ann	IR-TR
	*Echinopserinaceus* Kit Tan	Ch	Per	SA-SI
	*Launaeacapitata* (Spreng.) Dandy	Th	Ann	SA-AR
	*Launaeamassauensis (Fres.) Chiov.	Th	Ann	IR-TR + ME + SA-AR + SU-ZA
	*Launaeamucronata* Muschl.	Th	Ann	IR-TR + SA-SI
	*Launaeanudicaulis* (L.) Hook.f.	Ch	Ann	IR-TR + SA-SI + SU-ZA
	*Plucheadioscoridis* (L.) DC.	Ch	Per	SA-AR+SU-ZA
	*Reichardiatingitana* (L.) Pau	Th	Ann	IR-TR + ME + SA-AR + SU-ZA
	*Senecioflavus* (Decne.) Sch.Bip.	Th	Ann	IR-TR + ME + SA-SI
	*Sonchusoleraceus* L.	Th	Ann	COSM
	*Verbesinaencelioides (Cav.) A.Gray	Th	Ann	NEO
	*Volutariasinaica (DC.) Wagenitz	Th	Ann	SA-AR+SU-ZA
Boraginaceae	*Arnebiahispidissima* (Lehm.) A.DC.	Th	Ann	IR-TR + SA-AR + SU-ZA
	*Heliotropiumbacciferum* Forssk.	Ch	Per	IR-TR + SA-AR + SU-ZA
	*Heliotropiumcalcareum* Stocks	Ch	Per	IR-TR+SA-AR
	*Heliotropiumcurassavicum* L.	Th	Per	TR
	*Heliotropiumdigynum* (Forssk.) Asch. ex C.Chr.	Ch	Per	SA-AR
	*Trichodesmaafricanum* (L.) Sm.	Ch	Per	SA-AR+SU-ZA
Brassicaceae	*Anastaticahierochuntica* L.	Th	Ann	SA-AR
	*Diplotaxisharra* Boiss.	Th	Ann	SA-AR
	*Eremobiumaegyptiacum* (Spreng.) Asch. ex Boiss.	Th	Ann	SA-AR
	*Erucasativa* Mill.	Th	Ann	IR-TR + ME + SA-AR
	*Erucariahispanica* Druce	Th	Per	ME + SA-SI
	*Farsetialinearis* Decne. ex Boiss.	Th	Per	SA-AR+SU-ZA
	*Morettiaparviflora* Boiss.	Ch	Per	SA-AR+SU-ZA
	*Notocerasbicorne* Amo	Th	Ann	SA-AR+SU-ZA
	*Physorhynchuschamaerapistrum* Boiss.	Ch	Ann	IR-TR + SA-AR + SU-ZA
	*Savignyaparviflora* Webb.	Th	Ann	SA-AR
	*Sisymbriumirio* L.	Th	Ann	ME + SA-SI + SU-ZA
Caryophyllaceae	*Gymnocarpossclerocephalus* (Decne.) Dahlgren & Thulin	Ch	Per	SA-AR
	*Paronychiaarabica* (L.) DC.	He	Ann	IR-TR
	*Polycarpaearobbairea (Kuntze) Greuter & Burdet	He	Ann	SA-AR+SU-ZA
Cistaceae	*Helianthemumlippii* (L.) Dum.Cours.	Ch	Per	SA-SI + SU-ZA
Cleomaceae	*Cleomeamblyocarpa* Barratte & Murb.	Th	Ann	IR-TR + SA-AR + SU-ZA
	*Cleomepallida* Kotschy	Ch	Per	SA-AR+SU-ZA
	*Cleomerupicola*Vicary	Ch	Per	SA-AR
	*Cleomescaposa* DC.	Th	Ann	IR-TR + SA-AR + SU-ZA
Convolvulaceae	*Convovulusprostratus* Forssk.	Ch	Per	SA-AR+SU-ZA
	*Convolvulusvirgatus* Boiss.	Ch	Per	IR-TR + SA-SI
Cucurbitaceae	*Cucumisprophetarum* L.	He	Per	IR-TR + ME + SA-SI +SU-ZA
Cucurbitaceae	*Citrulluscolocynthis* (L.) Schrad.	He	Per	IR-TR + ME + SA-SI +SU-ZA
Cyperaceae	*Cyperusconglomeratus* Rottb.	Ge	Per	SA-AR
	*Cyperusrotundus* L.	Ge	Per	COSM
Euphorbiaceae	*Chrozophoraoblongifolia* (Delile) Spreng.	Ch	Per	SA-SI + SU-ZA
	*Euphorbiaprostrata* Aiton	Th	Ann	IR-TR + SA-SI + SU-ZA
Fabaceae	*Argyrolobiumroseum* (Cambess.) Jaub. & Spach	He	Ann	SA-SI + SU-ZA
	*Astragaluseremophilus* Boiss	Th	Ann	IR-TR + ME + SA-AR
	Astragalusvogeliisubsp.fatimensis Maire	Th	Ann	SA-AR
	*Crotalariaaegyptiaca* Benth.	Ch	Per	IR-TR + SA-AR + SU-ZA
	*Hippocrepisconstricta* Kunze	Th	Ann	ME+SA-AR
	*Indigoferaarabica* Burm.f.	Ch	Per	SU-ZA
	*Medicagosativa* L.	Th	Ann	IR-TR+SA-AR
	*Prosopiscineraria* (L.) Druce	Ph	Per	SA-SI
	*Rhynchosiaminima* (L.) DC.	Ch	Per	SA-AR
	*Sennaitalica* Mill.	Ch	Per	IR-TR + ME + SA-SI +SU-ZA
	*Tavernieraglabra* Boiss.	Ch	Per	IR-TR + SA-AR
	Tephrosiapurpureasubsp.apollinea (Delile) Hosni & El-Karemy	Ch	Per	SU-ZA
	*Tephrosianubica* (Boiss.) Baker	Ch	Per	SA-AR+SU-ZA
	*Vachelliatortilis* (Forssk.) Galasso & Banfi	Ph	Per	IR-TR + ME + SA-AR + SU-ZA
Frankeniaceae	*Frankeniapulverulenta* L.	Th	Ann	IR-TR+ME
Geraniaceae	*Monsoniaheliotropoides* Boiss.	Ch	Per	SA-AR+SU-ZA
	*Monsonianivea* Webb	Ch	Per	SA-AR+SU-ZA
Gisekiaceae	*Gisekiapharnaceoides* L.	Th	Ann	PAL
Lamiaceae	*Salviamacrosiphon Boiss.	Th	Ann	IR-TR+ME
Malvaceae	*Corchorusdepressus* (L.) Stocks	Th	Per	ME+SA-AR
	*Corchorustrilocularis* L.	Th	Ann	TR
	*Hibiscusmicranthus* L.f.	Th	Ann	SA-AR+SU-ZA
	*Malvaparviflora* L.	Th	Ann	IR-TR + ME + SA-SI
Molluginaceae	*Glinuslotoides L.	Th	Ann	IR-TR+ME+TR
Neuradaceae	*Neuradaprocumbens* L.	Th	Ann	COSM
Nyctaginaceae	*Boerhaviadiffusa* L.	Ch	Per	COSM
	*Boerhaviaeleganssubsp.stenophylla (Boiss.) A.G.Mill.	Th	Ann	SA-SI
Plantaginaceae	*Plantagoamplexicaulis* Cav.	Th	Ann	IR-TR + ME + SA-SI
	*Plantagoafra* L.	Th	Ann	IR-TR + ME + SA-SI
	*Plantagociliata* Desf.	Th	Ann	IR-TR + SA-AR + SU-ZA
Poaceae	*Aristidaadscensionis* L.	Th	Ann	IR-TR + SA-AR + SU-ZA
	*Cenchrusciliaris* L.	He	Per	SA-AR+SU-ZA
	*Cenchrusdivisus* (J.F.Gmel.) Verloove, Govaerts & Buttler	He	Per	SA-AR
	*Cenchrussieberianus* (Schltdl.) Verloove	He	Ann	SA-AR
	*Echinochloacolonum* (L.) Link	Th	Per	IR-TR+ME+TR
	*Panicumantidotale* Retz.	He	Per	SA-AR+SU-ZA
	*Stipagrostisplumosa* (L.) Munro ex T.Anderson	He	Per	IR-TR + SA-AR + SU-ZA
	*Centropodiaforskaolii* (Vahl) Cope	He	Ann	IR-TR+SA-AR
	*Cynodondactylon* (L.) Pers.	Th	Per	PAN
	*Panicumturgidum* Forssk.	He	Per	SA-AR+SU-ZA
	*Tragusberteronianus* Schult.	Th	Ann	SA-AR+SU-ZA
	*Tragusracemosus* (L.) All.	Th	Ann	SA-AR+SU-ZA
	*Tripidiumravennae* (L.) H.Scholz	Ge	Per	SA-AR+SU-ZA
	*Polygalaerioptera* DC.	Ch	Ann	SA-AR
Polygalaceae	*Polygalairregularis* Boiss.	Ch	Ann	SA-SI + SU-ZA
Polygonaceae	*Emexspinosus* L.	Th	Ann	PAN
	*Rumexvesicarius* L.	Th	Ann	ME+SA-AR+SU-ZA
Portulacaceae	*Portulacaoleracea* L.	Th	Ann	COSM
Resedaceae	*Ochradenusarabicus* Chaudhary, Hillc. & A.G.Mill.	Ch	Per	SA-AR
	*Oligomerislinifolia* (Vahl ex Hornem.) J.F.Macbr.	Ann	Th	IR-TR+ME
	*Resedaaucheri* Boiss.	Th	Per	IR-TR + SA-SI
Rhamanaceae	*Ziziphusspina-christi* (L.) Desf.	Ph	Per	IR-TR + ME + SA-AR + SU-ZA
Rubiaceae	*Kohautiacaespitosa* Schnizl.	Th	Ann	SU-ZA
Solanaceae	*Lyciumshawii* Roem. & Schult.	Ph	Per	IR-TR + SA-AR + SU-ZA
	*Solanumnigrum* L	Ch	Per	SA-AR+SU-ZA
Tamaricaceae	*Tamarix* sp.	Ph	Per	ME+SA-AR+SU-ZA
Urticaceae	*Forsskaoleatenacissima* L.	Ch	Per	IR-TR + ME + SA-SI +SU-ZA
	*Forsskaoleaviridis Ehrenb. ex Webb	Th	Ann	SA-AR+SU-ZA
Zygophyllaceae	*Tribulusarabicus* Hosni	He	Per	SA-AR
	*Tribulusmacropterus* Boiss.	He	Per	IR-TR + SA-SI
	*Tribuluspentandrus* Forssk.	He	Per	PAN
	*Tribulusterrestris* L.	Th	Ann	ME+SU-ZA
	*Zygophyllumindicum* (Burm.f.) Christenh. & Byng	Ch	Per	SA-AR
	*Zygophyllumsimplex* L.	Th	Ann	SA-AR+SU-ZA
